# The effect of *Cinnamomum cassia* extract on the pancreatic tissue of albino diabetic rats

**DOI:** 10.25122/jml-2024-0167

**Published:** 2025-02

**Authors:** Afrah Hameed Sultan

**Affiliations:** 1 Anatomy and Histology Unit, Basic Science Department, College of Medicine, Hawler Medical University, Kurdistan Region, Iraq

**Keywords:** alloxan, diabetic rats, *Cinnamomum cassia*, pancreatic tissues, metformin

## Abstract

*Cinnamomum cassia* (*C. cassia*) has antihyperglycemic properties. This study aimed to assess the hypoglycemic effects of the aqueous extract of *C. cassia* on the pancreatic tissue of diabetic rats, comparing histological and biochemical outcomes with those of metformin (MET) administration. A total of 42 male albino rats were divided into seven groups. Distilled water was given to healthy rats in the first group, whereas diabetic rats (DRs) induced by alloxan were treated with the same substance in the second and third groups. Rats with diabetes were given *C. cassia* treatment for 14 and 30 days in the fourth and fifth groups, whereas non-DRs received the same treatment in the sixth and seventh groups. Furthermore, MET was administered to four groups of DRs. Diabetic rats had reduced serum amylase levels and significantly increased blood glucose levels. Histological examination revealed thickening of the basement membrane in the islets of Langerhans blood channels and capillaries, as well as an increase in α- and δ-cell activity and a reduction in β-cell activity. However, administration of *C. cassia* aqueous extract caused significant alterations in most measured parameters, including increased serum amylase and decreased blood sugar levels. The daily use of *C. cassia* decreased glucose levels and induced a substantial increase in β-cell activity and a decrease in α-cell activity. Plant extracts have both regenerative and reparative properties.

## INTRODUCTION

Diabetes, the most prevalent metabolic disorder in modern times, is a condition that affects the metabolism of carbohydrates, fats, and proteins. It is caused by either decreased insulin production or increased resistance to its effects [1,2]. Diabetes affects glucose and lipid metabolism, making it a major risk factor for cardiovascular disease [3]. Presently, the global incidence of diabetes is estimated to be 537 (10.5%) million individuals. The estimated prevalence is projected to increase to 643 million (11.3%) in 2030 and 783 million (12.2%) in 2045 [4]. Studies have shown that every year, approximately 41 thousand new cases of type 2 diabetes are detected worldwide [5]. More focus must be paid to the rising incidence of diabetes, as well as the numerous complications and high death rates associated with the condition [6]. Diabetes control is important for preventing complications and reducing mortality [7]. New and old drugs can be useful and important in controlling diabetes [8]. Metformin (MET) is an antihyperglycemic drug that reduces blood glucose (BG) levels by inhibiting endogenous glucose production [9].

Folk medicine and ancient healing methods have used plant compounds with hypoglycemic properties worldwide [10,11]. The effectiveness of many plants used in traditional medicine as oral anti-hyperglycemic medicines has been demonstrated by several investigations. Although some theories exist on how plants function, the precise mechanism is unknown [12].

### *Cinnamomum cassia* (Darseen)

Cinnamon bark has been used in Chinese medicine since 2700 B.C. and is said to supplement essential energy and blood, treat fever, diarrhea, menstrual disorders, tone the kidney and spleen, and serve as an antioxidant. *Cinnamomum cassia* (*C. cassia*), commonly known as cassia cinnamon, was the first cinnamon species introduced to Europe [13] and has been extensively used in traditional medicine in Russia, China, and Korea for the treatment of diabetes mellitus [14]. Recent animal studies have shown that *C. cassia* has a dose-dependent effect on reducing blood glucose levels, with higher doses exerting more pronounced hypoglycemic effects. Researchers have identified a specific molecule that mimics insulin and is responsible for its hypoglycemic properties as well as normalizing lipid profiles [15].

Despite the promising results of *C. cassia* in lowering BG levels, there is a lack of comprehensive studies examining its direct effects on pancreatic tissue at both histological and biochemical levels. Furthermore, the comparative efficacy of water-based *C. cassia* extracts with standard antidiabetic treatments like metformin remains understudied. This gap highlights the need for rigorous scientific investigation to validate and understand the potential therapeutic benefits of *C. cassia* in the context of pancreatic health in diabetes. The objective of this study was to investigate the blood sugar-lowering effects of water-based extracts derived from *C. cassia* on the pancreatic tissues of male rats with diabetes. Moreover, this research aimed to evaluate the outcomes of histological and biochemical assessments with the administration of MET.

## MATERIAL AND METHODS

### Animals and housing

This controlled laboratory experiment was conducted in 2021 at Hawler Medical University. Male adult Wistar albino rats aged 8-12 weeks, weighing 200–290 g, were included in this study. The rats were housed at the College of Medicine animal housing under standard laboratory settings (12h light/12h dark cycle, 22 ± 2 °C) [16]. The animals were provided with a standard pellet diet (composition: 21% protein, 5% fat, 4% fiber, 8% ash, 1% calcium, 0.6% phosphorus, and 3.4% glucose) and tap water ad libitum [17].

### Preparation of the aqueous plant extracts

The clean, dried stem bark of *C. cassia* (Darseen) was collected during the peak dry season (late February to early April). The bark was ground into a fine powder using a Wiley mill and stored at room temperature until required. For extraction, 100 mg of dried powdered stem bark was put in 20 ml boiling water for 1 hour, then cooled and filtered to make an aqueous extract.

### Induction of experimental diabetes

Diabetes was induced by administering a single intraperitoneal injection of alloxan monohydrate (BDH Chemical Ltd. England) at a dosage of 100 mg/kg into the peritoneal cavity [18].

### Experimental design

A total of 42 rats were allocated into seven groups, each consisting of six rats, to investigate the impact of a water-based cinnamon extract over 14 and 30 days.

Group I: Healthy rats treated with distilled water.

Group II: Diabetic rats (DRs) were administered alloxan for 14 days and administered distilled water.

Group III: DRs treated with alloxan and administered distilled water for 30 days.

Group IV: DRs treated with *C. cassia* for 14 days.

Group V: DRs treated with *C. cassia* for 30 days.

Group VI: Non-DRs treated with *C. cassia* for 14 days.

Group VII: Non-DRs treated with *C. cassia* for 30 days.

The aqueous plant extract (100 mg/kg/day) was administered orally via gavage to groups IV, V, VI, and VII for 14 days and 30 days.

### Metformin

To evaluate the effects of metformin (MET), 24 rats were divided into four groups (*n* = 6 per group).

Group I: 14 days normal control rats + aqueous solution of MET

Group II: 14 days diabetic control rats + aqueous solution of MET

Group III: 30 days normal rats + aqueous solution of MET

Group IV: 30 days DRs + aqueous solution of MET

Metformin (100 mg/kg/day) was administered orally by gavage for 14 and 30 days. Body weight (BW) and fasting blood glucose (FBS) levels were measured weekly throughout the experiment.

### Sampling and biochemical analysis

At the end of 14 and 30 days, the rats underwent an overnight fasting period (12–18 hours) before euthanasia using chloroform anesthesia. Blood samples were obtained via cardiac puncture using a sterile plastic syringe.

### Preparation of tissue samples

Pancreatic samples were taken from the euthanized animals on days 14 and 30 following the oral administration of the plant extract. Each sample was fixed in Bouin's solution for 24 hours. The fixed tissues were subsequently embedded in paraffin wax and sectioned using an electronic microtome at a thickness of 5 µm. The sections were mounted onto glass slides and subjected to Periodic Acid Schiff (PAS) and immunohistochemistry (IHC) staining to assess insulin and glucagon expression.

### IHC staining method

Expression of insulin and glucagon in pancreatic tissues was detected using IHC staining. The procedure followed the Dako Cytomation EnVision+ Dual Link system with Horseradish Peroxidase (DAB+) detection.

### Histological measurements and immunostaining quantification

The depth of the basement membrane in pancreatic sections stained with PAS dye was measured using a calibration stage micrometer. Immunostaining results were evaluated based on diaminobenzidine (DAB) staining, which produces a distinct brown cytoplasmic signal in positively stained cells. Grid cell count, a special computerized process, was used to measure the cells. A total of 1,000 cells per sample were analyzed across ten high-power fields (HPFs). Cells exhibiting significant brown cytoplasmic staining were classified as immunopositive, and the immunostaining index was determined by calculating the proportion of positive cells relative to the total count. A minimum of ten HPFs were evaluated for each case to establish a scoring basis. Immunostaining was scored as follows.

Negative: <5%

Mild: >5% and <20%

Moderate: >20% and <50%.

Strong positive: > 50.

### Statistical analysis

Statistical analyses were conducted using IBM SPSS Statistics for Windows, Version 21.0 (Armonk, NY, IBM Corp). Data were expressed as mean ± standard error of the mean (M ± SEM). The data were analyzed using one-way analysis of variance (ANOVA). The groups were distinguished using Duncan's test. The threshold for statistical significance was fixed at *P* < 0.05. The experiments were authorized by the ethics committee of the Hawler Medical University College of Medicine.

## RESULTS

The BG levels of rats with alloxan-induced diabetes at the 14^th^ and 30^th^ intervals were significantly elevated. Compared with the diabetic control group (CG), rats treated with *C. cassia* extract (treated DRs) had a significant reduction in BG levels, whereas untreated diabetic rats (untreated DRs) showed no significant changes over the study period. Conversely, the levels of serum amylase and BW in rats with alloxan-induced diabetes were significantly diminished at the 14^th^ and 30^th^ time points, whereas a substantial increase was observed in the treated DRs when compared to the diabetic CG. Although there were no significant improvements in BW among the treated non-DRs at 14 and 30 days, there were significant increases in serum amylase at these time points compared to the CG, as demonstrated in Tables 1 and 2.

**Table 1:** Effects of C. cassia on BW and biochemical parameters of normal and DRs

Tests	I	VI	VII	II	IV	III	V
**Glucose (mg/dl)**	97.7 ± 6.68 a	111.3 ± 4.07 a	103 ± 4.53 a	248.33 ± 21.35 b	102.2 ± 11.07 a	303.8 ± 40.70 B	119.3 ± 3.98 a
**Amylase (IU/L)**	756.5 ± 66.77 b	1169.3 ± 68.16 c	959.1 ± 63.72 c	349 ± 31.73 a	681.2 ± 54.95 b	411.5 ± 36.02 a	786.6 ± 75.02 bc
**Body weight (gm)**	258.3 ± 6.1 c	270.8 ± 5.68 c	274.7 ± 8.72 c	206.5 ± 3.94 a	226.7 ± 4.01 b	191.7 ± 3.3 a	240.8 ± 3.00 b

* Significant differences at P <0.05 are indicated by different letters

**Table 2:** The effects of MET (100 mg/kg) on BW and biochemical parameters in normal and DRs

Tests	I	VI	VII	II	IV	III	V
**Glucose (mg/dl)**	97.7 ± 6.68 a	108.7 ± 3.94 a	114.3±4.79 a	248.33 ± 21.35 b	115.5 ± 3.56 a	303.8 ± 40.70 b	119.5 ± 19.43 a
**Amylase (IU/L)**	756.5 ± 66.77 b	842.6 ± 46.71 b	735 ± 91.12 b	349 ± 31.73 a	806.1 ± 64.53 b	411.5 ± 36.02 a	820.6 ± 126.57 b
**Body weight (gm)**	258.3 ± 6.1 c	269.2 ± 8.10 c	273.3 ± 6.72 c	206.5 ± 3.94 a	230 ± 5.3 b	191.7 ± 3.3 a	236.7 ± 4.01 b

**Figure 1:**
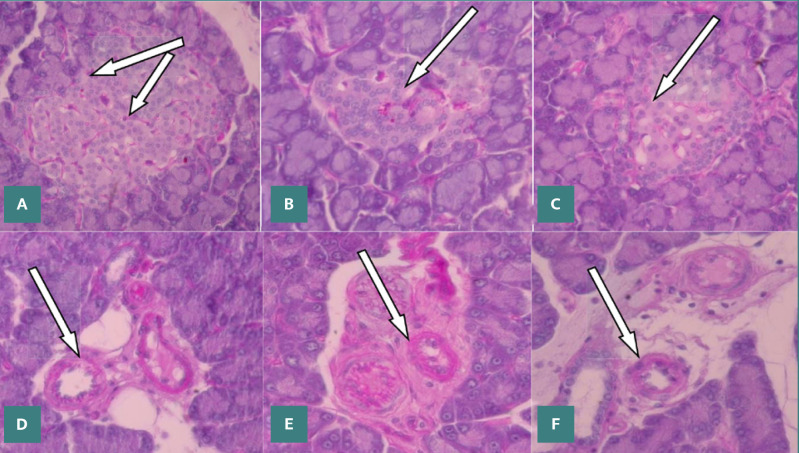
Microscopic view of rat pancreatic tissue. A, normal pancreas section shows few capillaries in the islets of Langerhans (arrows) stained with PAS at 1000x magnification. B, diabetic pancreas reveals a thickened islet capillary basement membrane (arrows) stained with PAS at 1000x. C, diabetic pancreas section, post-treatment with C. cassia extract, shows a thinner capillary basement membrane (arrow), stained with PAS at 1000x. D, normal pancreas section displays arterioles in the exocrine part with a clear basement membrane (arrow) stained with PAS at 1000x. E, diabetic pancreas shows a thickened arteriolar basement membrane (arrow), stained with PAS at 1000x. F, pancreatic section, post-treatment with C. cassia extract, showed a thinner arteriolar basement membrane (arrows), stained with PAS at 1000x.

Significant hypertrophy of the basement membrane was observed in the vascular and sinusoidal structures within the pancreatic islets at both 14- and 30-days post-induction of diabetes, as evidenced by the data presented in Tables 3 and 4 and Figure 1. The increase in membrane thickness was more pronounced at 30 days, particularly in the sinusoidal basement membranes, compared to measurements at 14 days.

**Table 3:** Effects of C. cassia on pancreatic histological parameters in normal and diabetic rats

	I	VI	VII	II	IV	III	V
**BV.BM** **(µm)**	2.96 ± 0.26 a	2.86 ± 0.10 a	2.73 ± 0.16 a	5.58 ± 0.42 c	3.93 ± 0.40 b	5.73 ± 0.30 c	2.86 ± 0.25 a
**C.BM** **(µm)**	0.27 ± 0.01 a	0.24 ± 0.01 a	0.24 ± 0.01 a	0.68 ± 0.07 b	0.30 ± 0.02 a	0.87 ± 0.10 c	0.27 ± 0.02 a
**α-cell %**	26.61 ± 1.59 a	26.66 ± 0.88 a	26.15 ± 0.70 a	86.33 ± 1.89 d	46.78 ± 1.41 c	87.53 ± 1.64 d	37.48 ± 1.72 b
**β-cell %**	71.15 ± 1.86 a	70.98 ± 1.23 a	71.70 ± 0.73 a	9.03 ± 1.52 d	50.31 ± 1.84 c	7.88 ± 1.63 d	59.93 ± 1.69 b
**δ-cell %**	2.23 ± 0.39 a	2.35 ± 0.66 a	2.15 ± 0.41 a	4.63 ± 0.38 b	2.90 ± 0.56 a	4.75 ± 0.35 b	2.73 ± 0.28 a
**IHC-insulin**	61.16 ± 4.11 a	59.16 ± 3.21 a	57.16 ± 2.54 a	7.66 ± 0.66 c	12.16 ± 1.75 b	8.50 ± 0.76 c	17.50 ± 0.76 b
**IHC-glucagon**	10.16 ± 1.24 a	10.33 ± 0.84 a	10.83 ± 1.01 a	58.33 ± 4.11 c	29.50 ± 3.15 b	65.00 ± 4.39 c	24.83 ± 1.81 b

**Table 4:** Effect of MET on the pancreatic histological alterations in normal and diabetic rats

	I	VI	VII	II	IV	III	V
**BV.BM** **(µm)**	2.96 ± 0.26 a	2.90 ± 0.11 a	2.93 ± 0.12 a	5.58 ± 0.42 c	3.95 ± 0.28 b	5.73 ± 0.30 c	3.35 ± 0.20 ab
**C.BM** **(µm)**	0.27 ± 0.01 a	0.25 ± 0.01 a	0.23 ± 0.01 a	0.68 ± 0.07 b	0.29 ± 0.02 a	0.87 ± 0.10 c	0.30 ± 0.01 a
**α-cell %**	26.61 ± 1.59 a	24.66 ± 1.69 a	27.61 ± 1.13 a	86.33 ± 1.89 d	46.06 ± 2.52 c	87.53 ± 1.64 d	39.91 ± 2.03 b
**β-cell %**	71.15 ± 1.86 a	73.40 ± 2.06 a	71.65 ± 1.55 a	9.03 ± 1.52 d	51.05 ± 2.66 c	7.88 ± 1.63 d	57.10 ± 2.07 b
**δ-cell %**	2.23 ± 0.39 a	1.93 ± 0.50 a	2.40 ± 0.31 a	4.63 ± 0.38 b	2.58 ± 0.48 a	4.75 ± 0.35 b	2.98 ± 0.89 a
**IHC-insulin**	61.16 ± 4.11 a	61.66 ± 3.80 a	59.33 ± 3.92 a	7.66 ± 0.66 b	12.00 ± 1.65 b	8.50 ± 0.76 b	16.00 ± 1.18 b
**IHC-glucagon**	10.16 ± 1.24 a	11.33 ± 1.64 a	12.16 ± 1.42 a	58.33 ± 4.11 c	34.00 ± 3.84 b	65.00 ± 4.39 c	33.83 ± 3.23 b

In contrast, intervention with *C. cassia* extract and MET in DRs led to a significant decrease in membrane thickness at the 14^th^ and 30^th^-day intervals compared to the diabetic control cohort. However, at 14 days, the vascular basement membrane thickness in the treated DRs remained elevated above the standard range, only returning to near-normal levels by 30 days. In contrast, treatment in non-diabetic rats (non-DRs) did not result in any statistically significant changes in membrane thickness at either time point compared to CG (Tables 3 and 4, Figure 1).

The proportion of β-cells within the islets of Langerhans in alloxan-induced DRs was significantly reduced at both the 14^th^ and 30^th^-day intervals in contrast to non-diabetic CG (Tables 3 and 4). Treatment with *C. cassia* extract and MET led to a significant recovery of β-cell percentages at these time points compared to untreated DRs; however, these levels remained below the normative range by 30 days. Conversely, the percentages of α- and δ-cells in the islets of DRs were significantly elevated during the same period. The combined treatment partially mitigated these alterations, but the percentage of α-cells did not return to normal by the 30^th^ day. In non-DRs, administration of the same treatment did not result in significant deviations from the percentage of CG cells.

**Figure 2:**
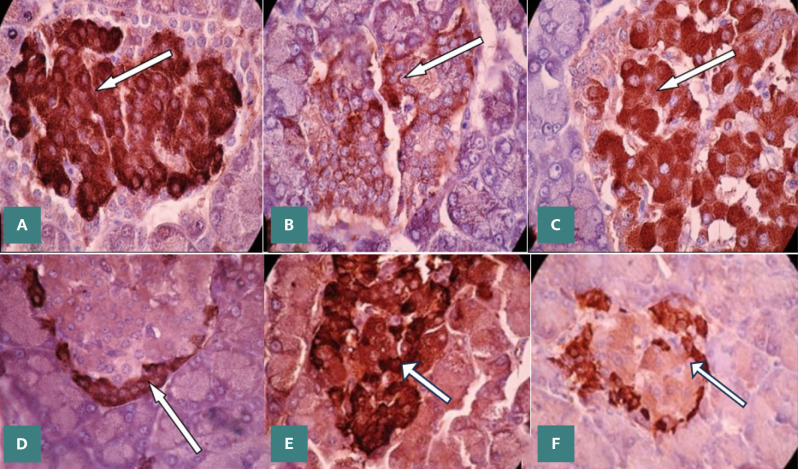
Immunohistochemical staining of pancreatic tissues in rats at 1000x magnification (IHC stain) using the DAP chromogen. A, panel displays normal pancreatic islets stained with anti-insulin antibodies, highlighting the presence of insulin-producing β-cells. B, the pancreas of DRs exhibited a notable decrease in insulin activity within β-cells. C, diabetic rat pancreatic islet post-treatment with C. cassia extract, showing partial recovery in β-cell activity. D, normal pancreas stained with anti-glucagon antibodies, indicating glucagon presence within α-cells. E, diabetic rat pancreatic islet reveals increased glucagon activity in α-cells. F, pancreatic islets of DRs after treatment with C. cassia extract, indicating a degree of normalization in α-cell activity.

Insulin immunoreactivity in alloxan-induced DRs was significantly reduced compared to the control cohort at 14 and 30 days, as shown in Tables 3 and 4 and Figure 2A-C. Treatment with *C. cassia* extract improved insulin immunoreactivity at the specified intervals, yet values did not reach those of the CG. Conversely, MET treatment did not significantly alter insulin levels at these time points compared to diabetic controls. Additionally, neither treatment (*C. cassia* extract or MET) induced significant changes in insulin immunoreactivity in the non-diabetic treated rats compared to controls at 14 and 30 days.

Tables 3 and 4 and Figure 2D-F illustrate that glucagon immunoreactivity was increased in DRs at 14 and 30 days compared to the CG. Treatment with *C. cassia* extract or MET resulted in a significant decrease in glucagon levels at the specified days in DRs, although not to the baseline levels of CG. No significant differences in glucagon immunoreactivity were observed when comparing non-DRs treated with both substances to control animals at the same periods.

## DISCUSSION

The BG levels in alloxan-induced diabetic rats showed a significant increase on the 14^th^ and 30^th^ days compared to the control group. This finding aligns with prior findings [19,20]. The observed increase in β-cell activity and decrease in α-cell activity in DRs treated with *C. cassia* also reflects the regenerative and reparative properties of plant extracts documented in previous studies. A study conducted by Fatima *et al*. that used nicotinamide-streptozotocin-induced non-obese type 2 DRs found that *C. cassia* extract modulated pancreatic β-cells and antioxidant status. Compared to the diabetic control group, the extract successfully brought the kidney function indicators, lipid profile, and plasma glucose levels back to normal. Following treatment with *C. cassia* extract, the histological analysis showed a significant reversal in the damage to pancreatic cells [21]. Other studies by Ayuob *et al*. [22] and Senevirathne *et al*. [15] showed similar results. Histological evaluation in the present study revealed an increase in β-cell populations, suggesting the possibility of β-cell regeneration or enhanced survival of partially damaged β-cells. This was accompanied by a significant decrease in the number of α- and δ-cells, likely as a result of β-cell recovery [23]. However, it is important to note that, despite these improvements, complete recovery was not achieved. The short duration of the treatment or the low concentration of insulin-like compounds in the plant extracts (which may need a longer time to become effective) could account for this.

The significant reduction in BG levels and increase in serum amylase activity observed in DRs treated with *C. cassia* are consistent with previous reports. For instance, a study by Ranasinghe *et al*. highlighted the hypoglycemic and antihyperlipidemic effects of *C. cassia* in both diabetic and non-diabetic models, supporting the current findings of improved glycemic control with *C. cassia* administration [24]. Similarly, another study by Qin *et al*. demonstrated that cinnamaldehyde, a major component of *C. cassia*, significantly lowered BG levels in diabetic mice, further corroborating our result [25]. In addition, the study of Raafat *et al*. confirms the results of the present study [26]. Plant extracts have a hypoglycemic impact that might be attributed to the existence of compounds similar to insulin in plants, notably flavonoids, which stimulate β-cell activation and regeneration, hence increasing the manufacture of insulin. Cinnamon extract controls insulin production and decreases serum glucose and cholesterol levels, according to in vivo, in vitro, and human studies [27,28].

Metformin, like the plant extract, induced a substantial drop in BG levels, as well as a rise in β-cells and a decrease in both α and δ cells in alloxan DRs. This result aligns with the findings of Naik *et al*. [29], who reported that MET increased pancreatic β-cell responsiveness to glucose and decreased gastrointestinal glucose absorption by reducing glucose toxicity and free fatty acid levels.

Compared to DRs, treated diabetic animals reported a substantial increase in BW on the 30th day of treatment with *C. cassia*. Plant extracts appear to have the capacity to partially restore hepatorenal damage, which explains their potential to increase BW. However, no significant changes in BW were noted at day 14, implying that longer treatment durations or higher extract concentrations may be required for sustained weight restoration. Similarly, MET also significantly improved BW in alloxan-treated DRs.

In this study, rats administered alloxan to induce diabetes had substantially lower blood amylase levels at 14 and 30 days than those in the CG. These results are consistent with those of Eleazu *et al*. [30] and Ebrahimi *et al*. [31], who found that diabetes impairs the activity of the exocrine pancreas and selectively impairs amylase secretion. Additionally, in this study, compared to diabetic controls, treatment with *C. cassia* extracts significantly increased serum amylase activity in DRs. The presence of pharmacologically active compounds in plants that promote insulin synthesis by β-cells and pancreatic exocrine function to release amylase may account for these results [32]. Metformin, like *C. cassia*, caused DRs to have significantly higher serum amylase levels. On days 14^th^ and 30^th^, there was a substantial decrease in α-cell activity and a non-significant difference in β-cell activity between DRs treated with MET and diabetic controls, as determined by IHC. However, in another study, MET was shown to increase insulin secretion and shield pancreatic cells against glucotoxicity and lipotoxicity [33-36].

The findings indicated that MET had no effect on β-cell regeneration. At 14- and 30 days following treatment with *C. cassia* and MET, no significant changes were seen in biochemical and histological analysis between treated non-DRs and the CG. These findings showed that administering *C. cassia* and MET to healthy animals did not impact their biochemical and histological changes.

### Limitations

The study's limitations include short duration, use of a single diabetes model, limited biochemical parameters, and lack of mechanistic insights and human data. Future research should involve larger, long-term studies, various diabetes models, comprehensive biochemical assessments, and clinical trials to confirm these findings and elucidate the mechanisms involved.

## CONCLUSION

This study demonstrates that treatment with *Cinnamomum cassia* extract and metformin significantly ameliorates several pathological changes in alloxan-induced DRs, including hyperglycemia, decreased serum amylase activity, and alterations in pancreatic cellular architecture. Notably, the interventions led to a reduction in BG levels and an increase in serum amylase levels, alongside marked improvements in the structural integrity of pancreatic tissues, as evidenced by the reduction in basement membrane thickness and a partial restoration of β-cell populations. These findings underline the potential of *C. cassia* extract as a complementary treatment for diabetes, offering benefits similar to established pharmaceuticals like metformin.

## Personal thanks

The author thanks the medical staff at the Hawler Medical University for their valuable assistance and support in conducting the laboratory research.
